# Confession of a Pediatric Oncologist

**DOI:** 10.1200/JGO.2016.009084

**Published:** 2017-04-27

**Authors:** Daniela Cristina Stefan

It was a beautiful sunny morning, many years ago. If you want the exact date, I could certainly find it somewhere in my medical notes and in the logbook that I was keeping at that time. It was one of the mornings when the sun was shining and the sky was blue. One could say it was just another typical day in Africa.

I was still a young doctor specializing in oncology, treating children with cancer and believing in miracles and the power of medicine. I remember clearly that invigorating morning as I was entering the white, long, cold corridor of the hospital where I worked in Cape Town, South Africa. That morning will stay with me for a long time, if not forever.

Sadly, that was the morning when I had to admit to myself, as a young doctor, that the science of medicine had failed us in the struggle to cure children with cancer. It was the same morning, without any clouds and with a bright shining sun, when I had to sit and explain to a child who was old enough to understand that we had reached the end of the road for her. We had reached the end of the road for hope, for smiles, and for the future. Those who know me may remember that I am not someone to give up hope easily or to accept defeat.

That was a special morning, one when I could not find my words easily, a morning when I was sweating and breathing fast with no apparent cause. I knew that I was overwhelmed by emotions and that this time was unlike any other for me.

I had arrived at the hospital earlier than usual and I was restless on my chair, wondering what I should say and not finding any words as I looked through the glass door at my patient. She was a tiny 16-year-old girl curled up in her bed, a young girl who used to have long and curly hair, big brown eyes, and a soft and gentle smile. For too many years, she had been fighting, with every fiber of her body, a lymphoma that was now on its way to winning the battle between them. I had been treating Natasha for a number of years, trying different protocols and treatment options. In a way, Natasha was another daughter of mine, for, as the time passed, I got to know her better and became closer to her.

That day deeply marked my career. It was that morning when I had to go in and confess to her that we had reached the end of the road. There was no more hope and all of her dreams—I knew them all—of studying to become a nurse, getting married, having kids, and playing guitar would remain unfulfilled.

How do you share such devastating news with a child, who is not only your patient but also a fellow human being with whom the pain, hope, sorrow, and eternity can be identified? It seemed that the moment did not care about my struggle with infinity and my own continuous wish to find words to explain the truth to her, not giving hope but alleviating the pain, the fear, and the unknown.

I approached her private room as slowly as I could. Her cubicle was just in front of the doctor’s station. I walked the few steps in several long minutes. Now it was just a sliding door that separated us. She could see me and, as she turned, she looked directly into my eyes. Her face lit up and she smiled openly. The joy of life was written all over her face.

I smiled too, or perhaps just grimaced as I pushed the sliding door open. There was no turning back. The door barely opened and I squeezed into the room, closing it carefully behind me, trying to delay the inevitable moment of telling her the truth.

I continued, not able to make a sound, as if my vocal cords had suddenly collapsed or become paralyzed. With a lump in my throat, I decided to sit on her bed, remembering from my third year as a student that I should never invade the private space of the patient. Yet there I was, barely breathing, with an empty mind, suffocated by my own uncontrollable feelings. I was powerless in the face of the disease and speechless in front of my patient who, without me noticing, had become my child too.

Then the unexpected happened. Natasha took my hand and said (I can still hear her clearly): “I am more worried about you. Me, I will be fine. You remain here to help the others; I will always be with you.”

That exemplified the wisdom and maturity of a 16-year-old girl, Natasha, who died later that day. Today I decided to write down this memory, as I had planned to do many times but never shared it with anyone.

The death of a child has a different significance for each of us. Childhood cancer should not be compared, nor should it have to compete, with any other form of cancer. No one has the right to talk about numbers, because there are no numbers. These are children—my children, your children, our children. They do not have loud voices to be heard. Rather, they express themselves most of the time by drawing; they listen to our stories.

Stop counting the number of children with cancer. Stop for a moment and try to imagine if that was your child…it is not imaginable. We have seen progress in Africa too, as awareness translates into earlier diagnosis and then better survival. Even if we are still far from the desired rates of survival, we are slowly getting there. For some, there may still be enough time, but there was not for Natasha.

I am writing as a tribute to Natasha’s hope and her selfless wish for other children to get better. As promised, I am still standing here and looking for help in fulfilling my promise to her: Help the others.

